# An analysis and systematic review of sarcopenia increasing osteopenia risk

**DOI:** 10.1371/journal.pone.0250437

**Published:** 2021-04-28

**Authors:** Zhaowei Teng, Yun Zhu, Xiaochao Yu, Jie Liu, Qing Long, Yong Zeng, Sheng Lu

**Affiliations:** 1 The Sixth Affiliated Hospital of Kunming Medical University, Yuxi, China; 2 Yunnan Key Laboratory of Digital Orthopedics, The First People’s Hospital of Yunnan Province, Kunming, China; Clinca Geriatrica, ITALY

## Abstract

Sarcopenia is a progressive generalized skeletal muscle disorder, which may increase the risk of osteopenia. The aim of this study was to systematically review studies on the association between sarcopenia and osteopenia by pooled analysis. The PubMed and Embase databases were searched from inception to October 2020 for studies focusing on the association between sarcopenia and osteopenia. Two reviewers independently extracted data and assessed study quality. A pooled analysis was performed to calculate odds ratios (ORs) and 95% confidence intervals (CIs) using random-effects models. Subgroup analysis was conducted to explore the source of heterogeneity and the stability of outcome. A total of 25 independent studies involving 47,744 participants fulfilled the inclusion criteria. Sarcopenia significantly increased the risk of osteopenia (OR, 2.08; 95% CI, 1.66–2.60); Sensitivity analyses indicated the outcome was stable. Subgroup analyses showed that sarcopenia significantly increased osteopenia risk in each subgroup. No evidence of publication bias among the studies existed. In this study, our findings showed that sarcopenia significantly increased the risk of osteopenia. Thus, we suggest that sarcopenia can be a predictor of osteopenia risk.

## Introduction

Sarcopenia is a muscle disorder involving depletion of skeletal muscle mass with a risk of adverse outcomes, such as physical disability and poor quality of life [1], is associated with many clinical conditions, such as cancer, diabetes, rheumatoid arthritis, and osteopenia [2–4]. Osteopenia, defined by the World Health Organization that is a t-score between -1 to -2.5, is a clinical term used to describe a decrease in bone mineral density [5]. Projections estimate that over 47 million Americans will be afflicted with osteopenia [5, 6]. Thus osteopenia is one of the major public health problems globally, and the burden is extremely heavy.

Some studies have indicated that osteopenia is associated with an increased risk of sarcopenia [4, 7–16]. However, others have shown no significant association exists between sarcopenia and osteopenia [17–19]. Therefore, we performed a pooled analysis to assess the relationship between sarcopenia and osteopenia risk.

## Methods

This analysis was conducted in accordance with the Meta-analysis of Observational Studies in Epidemiology guidelines and the Preferred Reporting Items for Systematic Reviews and Meta-analyses standards [20, 21].

### Search strategy and selection of eligible studies

We systematically searched PubMed and Embase (from their inception to October 1, 2020) for studies conducted on the association between sarcopenia and osteopenia. Our core search keywords are as follows: “sarcopenia”, “osteopenia”, and “low bone mineral density”. Two researchers (TZW and ZY) independently reviewed the titles and abstracts of the studies retrieved from the databases. We included studies that reported sufficient data on sarcopenia increasing osteopenia risk, such as risk estimates (relative risks [RRs], odds ratios [ORs]) with 95% confidence intervals (CIs). The studies were assessed based on the Strengthening the Reporting of Observational Studies in Epidemiology (STROBE) statement [20]. All disagreements were resolved by discussion with the corresponding authors.

### Data extraction and analysis

The data extraction and analysis were similar as our previous studies [22]. The following variables were recorded as: name of the first author, year of publication, region in which the study was performed, type of study design, sample size, participant gender and age, risk estimates with 95% CIs, adjustment factors. When one study included more than one trial, we pooled the trials and considered each trial an independent study. We computed a pooled OR and 95% CI. The Cochrane Q and I^2^ statistics were used to evaluate the statistical heterogeneity [23]. When the P value was < 0.1 and the I^2^ value was > 50%, the data were considered to be heterogeneous, and a random-effects model [24] was applied. To further explore the origin of heterogeneity and the stability of conclusion, we also performed subgroup analyses by sex, study design, study region, and criteria of sarcopenia. A sensitivity analysis was conducted to estimate the influence of each individual study on the pooled result. Begg’s test and Egger’s test were used to assess the potential publication bias [25, 26]. STATA version 12.0 (College Station, TX, USA) was used to analyze the data.

## Results

### Selected studies

**Fig 1:**
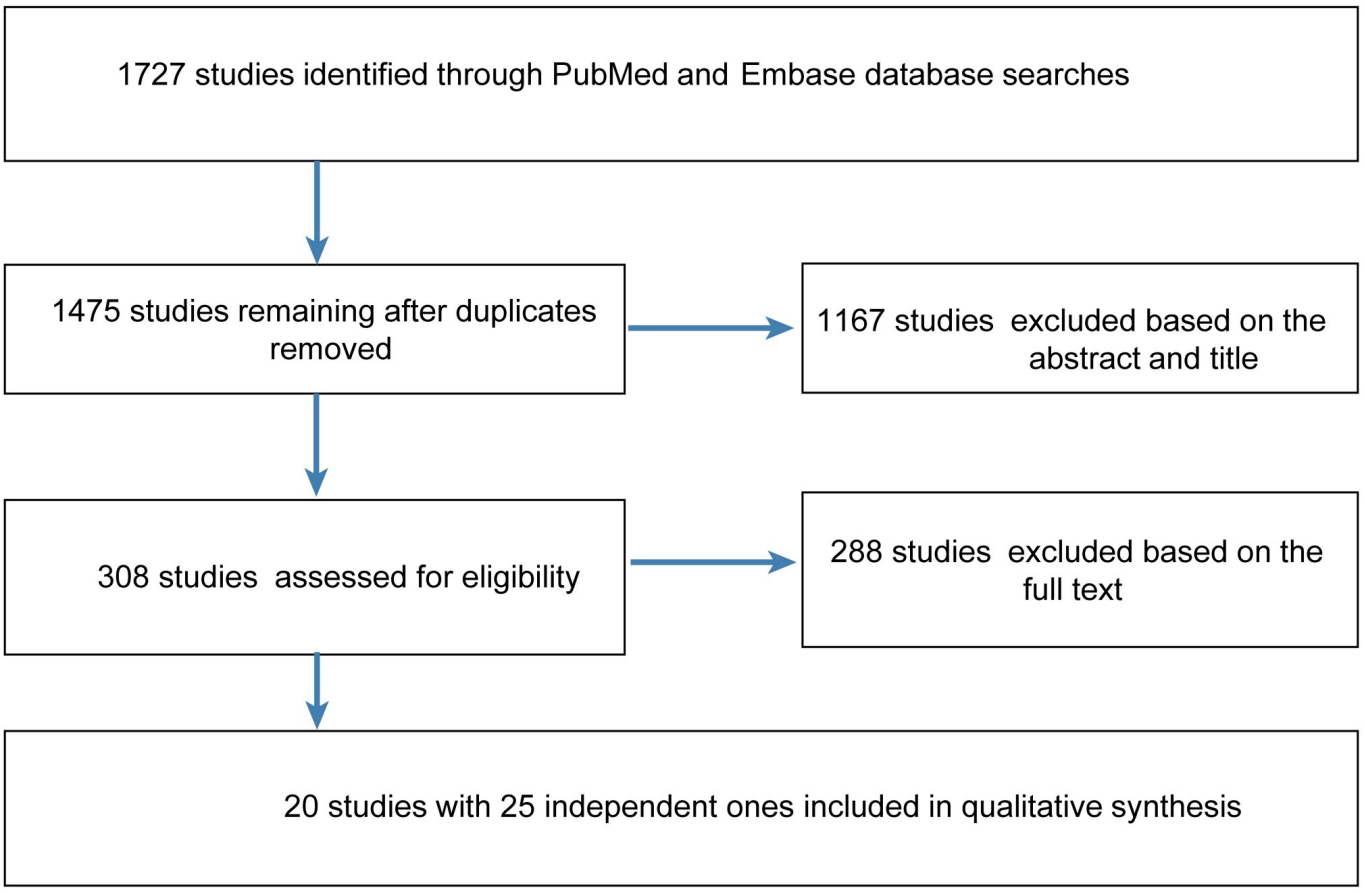
Flow diagram of the steps for study inclusion.

A total of 1727 studies were retrieved from PubMed and Embase, after removing duplicates, 1475 were identified. After screening the title and abstract, 288 necessitated reading of the full article. Ultimately, 20 studies [4, 7–19, 27–32] involving 47,744 participants were included (Fig 1). The study characteristics are listed in Table 1. The quality of the studies access by the STROBE statement (S1 Table).

**Table 1:** Characteristics of the 20 eligible studies.

Study, year	Age (years)	Gender	Study design	Region	Sample size	OR	LCI	UCI	Population	Statistical analysis method	Adjustment factors	Diagnostic criteria for osteopenia	Diagnostic criteria for sarcopenia
Schneider, 2008	36.2±13.9	Female and male	Case-control study	France	132	4.529	2.072	9.903	Crohn’s disease	Chi-square test	NA	Osteopenia: T-score for BMD (g/cm2) below -1.0	Sarcopenia: appendicular skeletal muscle index (ASMI) < 5.45 kg/m2 for women and < 7.26 for men
Falutz, 2013 (M)	18–75	Male	NA	Modena	1243	2.610	1.836	3.709	HIV patients	Chi-square test	NA	Analysis-determined differences between proportions of patients with osteoporosis and normal bone mineral density (BMD)	Baumgartner (<7.26 kg/height2 in males)
Falutz, 2013 (W)	15–70	Female	NA	Modena	724	2.626	1.589	4.341	HIV patients	Chi-square test	NA	Analysis-determined differences between proportions of patients with osteoporosis and normal BMD	Baumgartner (<5.45 kg/height2 in females)
Lee, 2013 (M)	≥60	Male	Cross-sectional study	Korea	1596	1.680	1.220	2.330	KNHANES (2009–2010) participants	Multivariable logistic regression analysis	Regular exercise, smoking status, alcohol consumption, and vitamin supplementation	T-score<−1.0	Sarcopenia: <1 SD below the sex-specific mean for a young reference group
Lee, 2013 (W)	≥60	Female	Cross-sectional study	Korea	1886	1.430	1.120	1.820	KNHANES (2009–2010) participants	Multivariable logistic regression analysis	Regular exercise, smoking status, alcohol consumption, and vitamin supplementation	T-score<−1.0	Sarcopenia: <1 SD below the sex-specific mean for a young reference group
Wu, 2013	40–85	Female and male	Cross-sectional study	Taiwan	600	1.720	1.090	2.720	Ambulatory and healthy volunteers	Multiple logistic regression analysis	Age, gender, BMI group, exercise, antiosteoporotic agent use, vitamin/mineral supplement use, menopause, and HRT	T-score <-1.0	SMI < 8.87 and < 6.42 kg/m2 in Taiwanese men and women, respectively
Bryant, 2015	18–50	Female and male	Cross-sectional study	Australia	137	6.300	1.400	27.900	Irritable bowel disease (IBD) patients	Multivariate logistic regression analysis	NA	T-score of either site: -1 to -2.5	Sarcopenia: both ASMI and grip strength (GS) ≥1 SD below population mean
Pereira, 2015	68.3±6.8	Male	Cross-sectional study	Brazil	198	9.000	2.088	38.787	Healthy men	Regression analyses	Age and weight	Abnormal BMD for men: T-score < -1.0	EWGSOP: RASM <7.26 kg/m2 +low muscle strength or low physical performance (walking speed <1.0 m/s)
Chung, 2016	≥50	Female and male	Cross-sectional study	Korea	2344	1.069	0.791	1.444	KNHANES V (2010) participants	Multivariable logistic regression analysis	Age, sex, household income, current smoking status, alcohol consumption, vitamin D, hypertension and dyslipidemia	T-score < -1.0	Sarcopenia: SMI score in the fifth percentile of sex-matched younger (20–40 years of age) reference KNHANES V-1 participants; SMI cutoff values: 28.9% for men and 22.4% for women
He, 2016	18–97.5	Female and male	Cross-sectional study	USA	17891	1.87	1.09	3.20	Chinese individuals African American individuals Caucasian individuals	Multivariate logistic regression analysis	Age, gender, height, weight, race, city, smoking, alcohol drinking, and regular exercise	WHO diagnostic classification: T-score < -1 SDs	Sarcopenia: (1) 6.08 and 4.79 kg/m2 for healthy Chinese men and women, respectively; (2) RASM ≤7.26 kg/m2 and RASM ≤5.45 kg/m2 in men and women, respectively, plus either low muscle strength or low physical performance
Lee, 2016	≥50	Female and male	Cross-sectional study	Korea	858	3.495	2.315	5.278	KNHANES IV, V (2008–2011) participants with chronic obstructive pulmonary disease (COPD)	Multivariate logistic regression analysis	Age; gender; height; smoking frequency; blood levels of vitamin D, parathyroid hormone (PTH), and alkaline phosphatase (ALP); forced expiratory volume in 1 second (FEV1, %); and physical inactivity level	WHO diagnostic classification: T-score < -1 SD	AWGS; sarcopenia: ASMI by DXA ≤7.0 kg/m2 for male patients and ≤ 5.4 kg/m2 for female patients
Lee Ih, 2016	≥65	Female	Cross-sectional study	Korea	269	1.240	0.583	3.210	Postmenopausal women living in local community centers	Binary logistic regression analyses	Age, postmenopausal period, body fat level and physical activity	Osteopenia: BMD > 1.0 but < 2.5 SDs below the young adult mean	Sarcopenia: weight-adjusted ASM < -2 SDs
Lee Ih, 2016	73.1 ± 5.5	Female	Cross-sectional study	Korea	119	4.420	0.962	20.315	Elderly women	Logistic regression analyses	Age	Osteopenia: -1.0 ≥ T-score > -2.5	ASMI < 5.27 kg/m2
Choi, 2017 non-TB	≥50	Male	Cross-sectional study	Korea	2699	3.283	2.690	4.007	KNHANES (2008–2011) participants	Chi-square test	NA	osteopenia: T-score between -2.5 and -1	Sarcopenia: ASMI cutoff of 6.96 kg/m2
Choi, 2017 (TB1)	≥50	Male	Cross-sectional study	Korea	98	7.448	2.488	22.299	Tuberculosis (TB) survivors among KNHANES (2008–2011) participants	Chi-square test	NA	osteopenia: T-score between -2.5 and -1	Sarcopenia: ASMI cutoff of 6.96 kg/m2
Choi, 201 (TB2)	≥50	Male	Cross-sectional study	Korea	245	3.268	1.855	5.757	TB survivors among KNHANES (2008–2011) participants	Chi-square test	NA	osteopenia: T-score between -2.5 and -1	Sarcopenia: ASMI cutoff of 6.96 kg/m2
Choi, 2017 (TB3)	≥50	Male	Cross-sectional study	Korea	186	5.115	2.452	10.672	TB survivors among KNHANES (2008–2011) participants	Chi-square test	NA	Osteopenia: T-score between -2.5 and -1	Sarcopenia: ASMI cutoff of 6.96 kg/m2
França, 2017	≥50	Female and male	Cross-sectional study	Brazil	214	2.410	1.070	5.400	Health Survey-Sao Paulo (ISA-Capital2014/2015) participants	Logistic regression analysis	Age, sex and 25-hydroxyvitamin D (25OHD) levels	T-score <-1.0	ASM (sum of muscle mass of arms and legs, kg) divided by height2 (m2) classified according to the EWGSOP
Harris, 2017	50–79	Female	Observational and clinical trials	USA	10937	1.421	1.289	1.566	Women’s Health Initiative (WHI)- enrolled women	Chi-square test	NA	T-score <-1.0	Sarcopenia: 20th percentile of the distribution of residuals from a model in which aLM was corrected for fat mass and height and linear regression was performed to model the associations between aLM and (meters) and between aLM and fat mass (kg)
Hwang, 2017	63.9 ± 10.6	Male	Cross-sectional study	Korea	777	3.898	1.270	11.957	Male KNHANES (2008–2011) participants with COPD	Logistic regression analyses	NA	WHO criteria (T-score between -2.5 and -1)	Sarcopenia: ASMI < 2 SDs
Kim, 2017 (M)	25–49	Male	Cross-sectional study	Korea	1702	0.953	0.719	1.264	KNHANES IV, V (2008–2011) participants	Univariable logistic regression analysis	Confounders	T-score < -1.0	Severely low muscle mass: SMI >2 SDs below the gender-specific mean of the younger reference group (SMI [%]) calculated as the ASM (kg)/weight (kg) ×100
Kim, 2017 (W)	20–55	Female	Cross-sectional study	Korea	2192	0.843	0.665	1.068	KNHANES IV, V (2008–2011) participants, premenopausal women	Multivariable logistic regression analysis	Age, BMI, smoking, drinking, hypertension, physical activity, and serum 25OHD levels	T-score < -1.0 at the lumbar spine, femoral neck, and/or total hip	Severely low muscle mass: SMI >2 SDs below the gender-specific mean of the younger reference group (SMI [%]) calculated as the ASM (kg)/weight (kg) ×100
Magdalena, 2017	50–75	Female	Case-control study	Poland	51	3.778	1.144	12.472	Psoriatic arthritis patients	Chi-square test	NA	WHO diagnostic classification: T-score -1 to -2.5 SDs	Baumgartner et al.: aLM index < 5.45 kg/m2
Lee, 2017 (ACOS)	≥50	Female and male	Cross-sectional study	Korea	110	6.935	1.194	44.272	KNHANES IV, V (2008–2011) participants with asthma-COPD overlap syndrome (ACOS)	Multivariate logistic regression analysis	Age; gender; height; smoking frequency; blood levels of vitamin D, PTH, and ALP; FEV1 (%); and physical inactivity level	WHO diagnostic classification: T-score −1 to −2.5 SDs	AWGS; sarcopenia: ASMI by DXA ≤7.0 kg/m2 for male patients and ≤ 5.4 kg/m2 for female patients
Lee, 2017 (COPD)					748	3.131	2.101	4.666					
Lee, 2017 (Asthma)					89	0.268	0.043	1.684					
Santos, 2018	80–95	Female and male	Cross-sectional study	Brazil	128	2.810	1.110	7.110	Elderly residents	Binary logistic regression analysis	Gender, age and smoking	WHO diagnostic classification: T-score < -1.0 SD	aLM index < 7.59 kg/m2 and < 5.57 kg/m2 for men and women, respectively, with a gait speed < 0.8 m/s in a 3 m walking test
Bieliuniene, 2019	22–89	Female and male	Prospective study	Lithuanian	100	2.648	1.123	6.243	Patients with chronic pancreatitis (CP) and pancreatic ductal adenocarcinoma	Chi-square test	NA	Osteopenia: T-score from -1 to -2.5	Sarcopenia: SMI < 34.4 cm2/m2 for females and < 45.4 cm2/m2 for males

NA: not available; OR: estimate of the risk; LCI: low limit of 95% confidence interval; UCI: upper limit of 95% confidence interval.

### Main analysis

**Fig 2:**
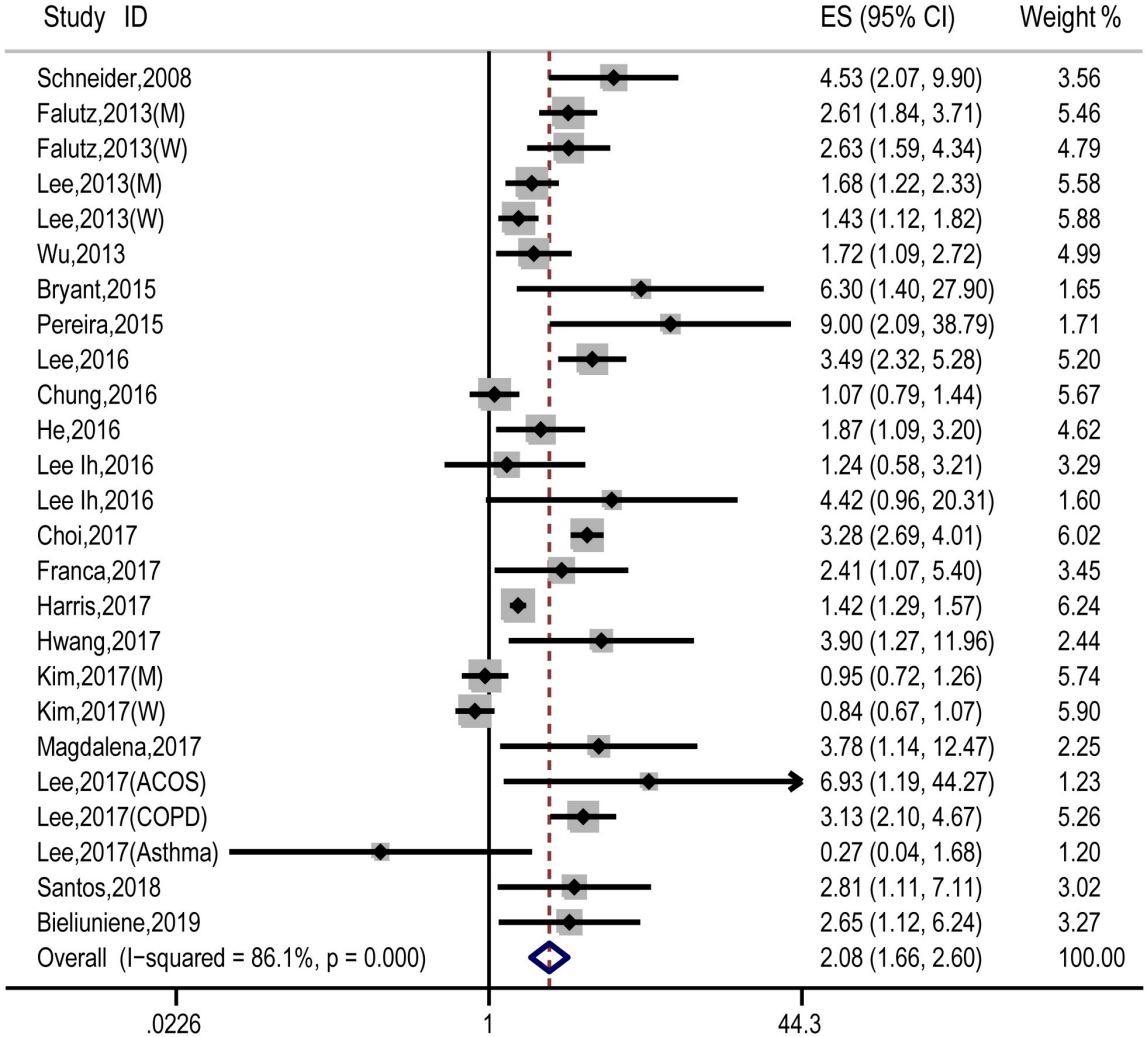
Forest plot of the estimated effects of sarcopenia on osteopenia risk.

**Fig 3:**
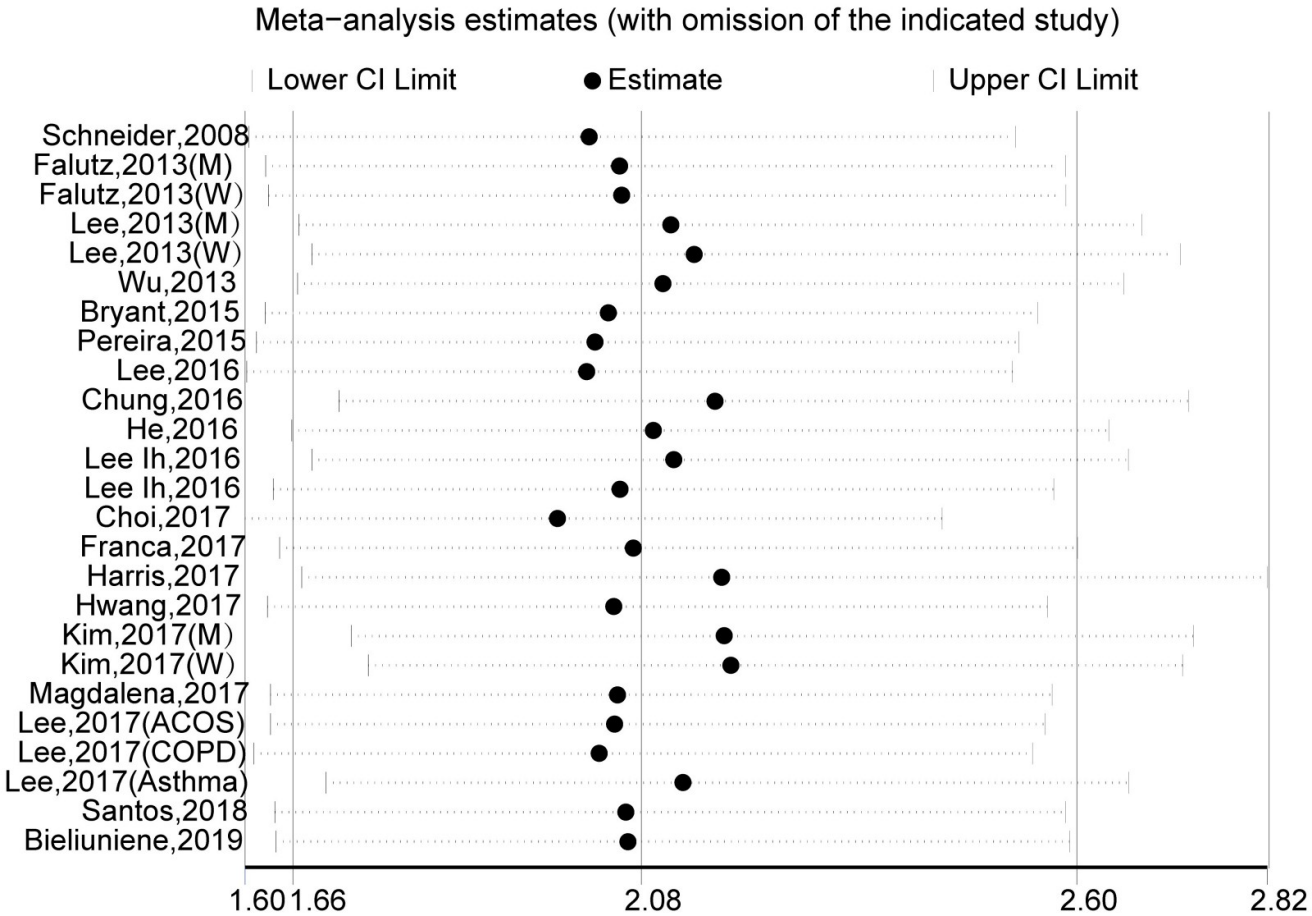
Sensitivity analysis for the estimated effects of sarcopenia on osteopenia risk. The analysis was performed via recalculation of the pooled results of the primary analysis after exclusion of one study per iteration.

**Fig 4:**
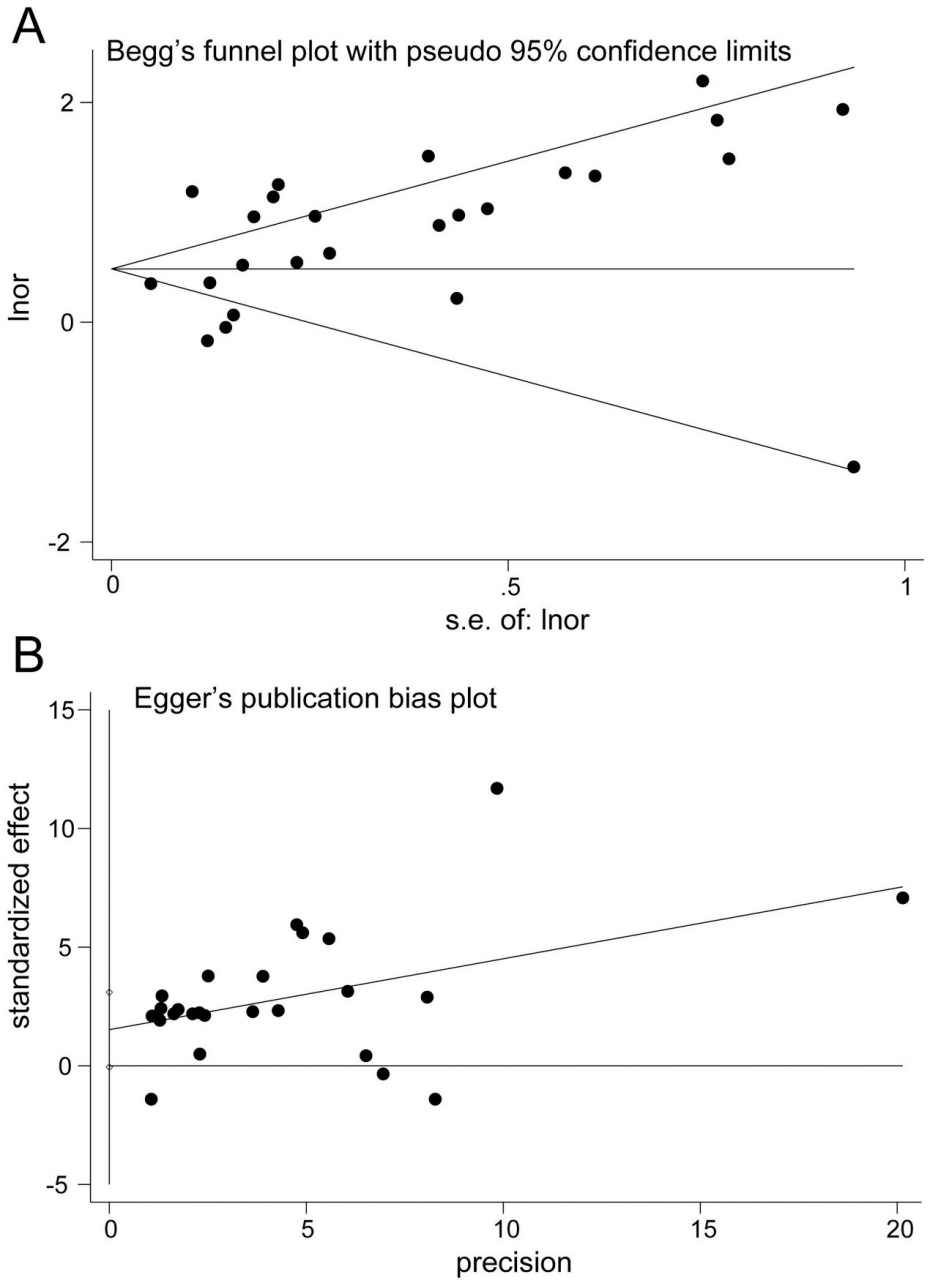
Publication bias plot. A, Begg’s funnel plot. B, Egger’s publication bias plot.

A pooled analysis of 20 studies involving 25 researches showed that sarcopenia significantly increased osteopenia risk (OR, 2.08 [95% CI, 1.66–2.60]; P_heterogeneity_ = 0.000, I^2^ = 86.1%) (Fig 2). Substantial heterogeneity was observed (P<0.10, I^2^ >50%) (Fig 2); however, the analysis revealed that exclusion of any single study did not alter the overall combined results, which indicated that the outcome was stable (Fig 3). Subgroup pooled analyses performed according to gender, study design type, different criteria of sarcopenia, and region also indicated that sarcopenia significantly increased osteopenia risk in each subgroup (Table 2). The Begg and Egger test indicated no evidence of publication bias among the studies [Begg, P > |z| = 0.168; Egger, P = 0.058, 95% CI -0.055–3.098] (Fig 4).

**Table 2:** Subgroup analysis for sarcopenia and risk of osteopaenia using random-effects model.

Factor	No.	OR (95% CI)	I^2^ (%)	P_heterogeneity_
**Study design**				
Cross-sectional study	19	1.99 (1.47, 2.70)	87.5	0.000
Case-control study	2	4.29 (2.23, 8.25)	0.0	0.803
Other	4	2.14 (1.37, 3.32)	82.4	0.001
**Sex**				
Male	6	2.36 (1.39, 4.02)	91.3	0.000
Female	7	1.49 (1.11, 2.00)	78.8	0.000
Male and female	12	2.37 (1.64, 3.44)	74.4	0.000
**Sarcopenia criteria**				
1- Ba 1-Baumgartner’s	7	2.45 (1.74, 3.44)	82.8	0.000
2- EWGSOP	5	2.39 (1.52, 3.78)	42.1	0.141
3- AWGS	4	2.83 (1.56, 5.15)	62.1	0.048
4-Others	9	1.34 (1.02, 1.75)	76.3	0.000
**Region**				
1-Europe	5	2.82 (2.19, 3.62)	0.0	0.750
2-America	4	2.41 (1.25, 4.64)	68.8	0.022
3-Asia	15	1.78 (1.28, 2.46)	89.5	0.000
4-Australia	1	6.30 (1.41, 28.12)	-	-

OR: estimate of the risk; CI: 95% confidence interval; No.: number of the included studies.

## Discussion

Osteopenia is characterized by loss of bone mass, reduced bone mineral density, which will develop into osteoporosis, may further lead to heavy economic and social burdens. Sarcopenia is one of the most important contributing factors related to osteopenia. Muscle and bone are interconnected biochemically and biomechanically, and they can mutually influence each other [33, 34]. Sarcopaenia and osteopaenia are two musculoskeletal pathologies mutually influencing each other, both associated with aging, lifestyle factors, falls and fractures [1, 3]. Thus, sarcopenia and osteopaenia frequently occur concomitantly, which leads to osteosarcopenia, and all of these conditions are critically associated with bone fragility, increased fall risk, fractures [35]. And osteosarcopaenia should be consciously incorporated into daily life and therapeutic strategies. This pooled analysis indicated that sarcopenia significantly increased osteopenia risk. Although heterogeneity was substantial, sensitivity analysis did not alter the overall combined results, subgroup analyses showed that sarcopenia significantly increased the risk of osteopenia in each pooled subgroup, which all demonstrated the credibility of the results. This pooled analysis has strengthened previous findings, for example, one study showed that older women with sarcopenia exhibited lower bone mineral density than those without sarcopenia [35]. Therefore, it may be possible to prevent osteopenia and related adverse events by the treatment of sarcopenia.

This study has several limitations. First, the study design included cross-sectional studies, case-control studies, and others, which might have led to substantial heterogeneity. Second, some trials did not provide the data as estimates with 95% CIs, so we had to calculate these values according to specific numbers of participants, which might have influenced the accuracy of the results. Third, different studies used different diagnostic criteria for sarcopenia, which might have slightly affected the results. Therefore, the results should be interpreted with caution.

## Conclusion

In this study, our findings showed that sarcopenia significantly increases osteopenia risk. However, care should be taken when interpreting the findings, and large randomized controlled trials are still needed to further specify the association between osteopenia and sarcopenia.

## Supporting information

S1 Table Methodological quality of studies included in the final analysis based on STROBE statement checklists. (PDF)

S1 Checklist PRISMA 2009 checklist. (PDF)

## References

[1] Cruz-Jentoft AJ, Sayer AA. Sarcopenia. Lancet. 2019;393(10191):2636–46. Epub 2019/06/07. 10.1016/S0140-6736(19)31138-9 . 31171417

[2] Kanis JA, Cooper C, Rizzoli R, Reginster JY, Scientific Advisory Board of the European Society for Clinical and Economic Aspects of Osteoporosis (ESCEO), The Committees of Scientific Advisors and National Societies of the International Osteoporosis Foundation (IOF). European guidance for the diagnosis and management of osteoporosis in postmenopausal women. Osteoporos Int. 2019;30(1):3–44. 10.1007/s00198-018-4704-5 . 30324412 PMC7026233

[3] Compston JE, McClung MR, Leslie WD. Osteoporosis. Lancet (London, England). 2019;393(10169):364–76. Epub 2019/01/31. 10.1016/s0140-6736(18)32112-3 . 30696576

[4] Bieliuniene E, Brondum Frokjaer J, Pockevicius A, Kemesiene J, Lukosevicius S, Basevicius A, et al. CT- and MRI-Based Assessment of Body Composition and Pancreatic Fibrosis Reveals High Incidence of Clinically Significant Metabolic Changes That Affect the Quality of Life and Treatment Outcomes of Patients with Chronic Pancreatitis and Pancreatic Cancer. Medicina (Kaunas, Lithuania). 2019;55(10). Epub 2019/10/02. 10.3390/medicina55100649 . 31569661 PMC6843405

[5] Varacallo M, Seaman TJ, Jandu JS, Pizzutillo P. Osteopenia. StatPearls. Treasure Island (FL): StatPearls Publishing Copyright © 2020, StatPearls Publishing LLC.; 2020. 29763053

[6] Varacallo MA, Fox EJ, Paul EM, Hassenbein SE, Warlow PM. Patients’ response toward an automated orthopedic osteoporosis intervention program. Geriatric orthopaedic surgery & rehabilitation. 2013;4(3):89–98. Epub 2013/12/10. 10.1177/2151458513502039 . 24319621 PMC3848331

[7] Santos VRD, Christofaro DGD, Gomes IC, Junior IFF, Gobbo LA. Relationship between obesity, sarcopenia, sarcopenic obesity, and bone mineral density in elderly subjects aged 80 years and over. Revista brasileira de ortopedia. 2018;53(3):300–5. Epub 2018/06/13. 10.1016/j.rboe.2017.09.002 . 29892580 PMC5993911

[8] Schneider S, Al-Jaouni R, Filippi J, Wiroth JB, Zeanandin G, Arab K, et al. Sarcopenia is prevalent in patients with Crohn’s disease in clinical remission. Inflammatory bowel diseases. 2008;14(11):1562–8. 10.1002/ibd.20504 18478564

[9] Falutz J, Rosenthall L, Guaraldi G. Association of osteoporosis and sarcopenia in treated HIV patients. Antiviral Therapy. 2013;18:A17.

[10] Lee SG, Lee YH, Kim KJ, Lee W, Kwon OH, Kim JH. Additive association of vitamin D insufficiency and sarcopenia with low femoral bone mineral density in noninstitutionalized elderly population: the Korea National Health and Nutrition Examination Surveys 2009–2010. Osteoporosis international: a journal established as result of cooperation between the European Foundation for Osteoporosis and the National Osteoporosis Foundation of the USA. 2013;24(11):2789–99. Epub 2013/05/09. 10.1007/s00198-013-2378-6 . 23652463

[11] Wu CH, Yang KC, Chang HH, Yen JF, Tsai KS, Huang KC. Sarcopenia is related to increased risk for low bone mineral density. Journal of clinical densitometry: the official journal of the International Society for Clinical Densitometry. 2013;16(1):98–103. Epub 2012/09/15. 10.1016/j.jocd.2012.07.010 . 22975297

[12] Bryant RV, Ooi S, Schultz CG, Goess C, Grafton R, Hughes J, et al. Low muscle mass and sarcopenia: common and predictive of osteopenia in inflammatory bowel disease. Alimentary pharmacology & therapeutics. 2015;41(9):895–906. Epub 2015/03/11. 10.1111/apt.13156 . 25753216

[13] Pereira FB, Leite AF, de Paula AP. Relationship between pre-sarcopenia, sarcopenia and bone mineral density in elderly men. Archives of endocrinology and metabolism. 2015;59(1):59–65. Epub 2015/05/01. 10.1590/2359-3997000000011 . 25926116

[14] Chung SM, Hyun MH, Lee E, Seo HS. Novel effects of sarcopenic osteoarthritis on metabolic syndrome, insulin resistance, osteoporosis, and bone fracture: the national survey. Osteoporosis international: a journal established as result of cooperation between the European Foundation for Osteoporosis and the National Osteoporosis Foundation of the USA. 2016;27(8):2447–57. Epub 2016/05/15. 10.1007/s00198-016-3548-0 . 27177746

[15] He H, Liu Y, Tian Q, Papasian CJ, Hu T, Deng HW. Relationship of sarcopenia and body composition with osteoporosis. Osteoporosis international: a journal established as result of cooperation between the European Foundation for Osteoporosis and the National Osteoporosis Foundation of the USA. 2016;27(2):473–82. Epub 2015/08/06. 10.1007/s00198-015-3241-8 . 26243357

[16] Lee DW, Choi EY. Sarcopenia as an Independent Risk Factor for Decreased BMD in COPD Patients: Korean National Health and Nutrition Examination Surveys IV and V (2008–2011). PloS one. 2016;11(10):e0164303. Epub 2016/10/18. 10.1371/journal.pone.0164303 . 27749901 PMC5066961

[17] Lee I, Cho J, Jin Y, Ha C, Kim T, Kang H. Body Fat and Physical Activity Modulate the Association Between Sarcopenia and Osteoporosis in Elderly Korean Women. Journal of sports science & medicine. 2016;15(3):477–82. Epub 2016/11/03. . 27803626 PMC4974860

[18] Lee I, Ha C, Kang H. Association of sarcopenia and physical activity with femur bone mineral density in elderly women. Journal of exercise nutrition & biochemistry. 2016;20(1):23–8. Epub 2016/06/15. 10.20463/jenb.2016.03.20.1.8 . 27298809 PMC4899897

[19] Kim IJ, Kang KY. Low Skeletal Muscle Mass is Associated with the Risk of Low Bone Mineral Density in Urban Dwelling Pr emenopausal Women. Calcified tissue international. 2017;101(6):581–92. 10.1007/s00223-017-0314-z . 28828511

[20] Vandenbroucke JP, von Elm E, Altman DG, Gotzsche PC, Mulrow CD, Pocock SJ, et al. Strengthening the reporting of observational studies in epidemiology (STROBE): explanation and elaboration. PLoS Med. 2007;4(10):e297. 10.1371/journal.pmed.0040297 . 17941715 PMC2020496

[21] Stroup DF, Berlin JA, Morton SC, Olkin I, Williamson GD, Rennie D, et al. Meta-analysis of observational studies in epidemiology: a proposal for reporting. Meta-analysis Of Observational Studies in Epidemiology (MOOSE) group. Jama. 2000;283(15):2008–12. 10.1001/jama.283.15.2008 . 10789670

[22] Teng Z, Zhu Y, Wu F, Zhu Y, Zhang X, Zhang C, et al. Opioids contribute to fracture risk: a meta-analysis of 8 cohort studies. PloS one. 2015;10(6):e0128232. Epub 2015/06/02. 10.1371/journal.pone.0128232 . 26030421 PMC4452583

[23] Higgins JP, Thompson SG, Deeks JJ, Altman DG. Measuring inconsistency in meta-analyses. BMJ. 2003;327(7414):557–60. 10.1136/bmj.327.7414.557 . 12958120 PMC192859

[24] DerSimonian R, Laird N. Meta-analysis in clinical trials. Control Clin Trials. 1986;7(3):177–88. 10.1016/0197-2456(86)90046-2 . 3802833

[25] Begg CB, Mazumdar M. Operating characteristics of a rank correlation test for publication bias. Biometrics. 1994;50(4):1088–101. . 7786990

[26] Egger M, Davey Smith G, Schneider M, Minder C. Bias in meta-analysis detected by a simple, graphical test. Bmj. 1997;315(7109):629–34. Epub 1997/10/06. 10.1136/bmj.315.7109.629 . 9310563 PMC2127453

[27] Choi CJ, Choi WS, Kim CM, Lee SY, Kim KS. Risk of Sarcopenia and Osteoporosis in Male Tuberculosis Survivors: Korea National Health and Nutrition Examination Survey. Scientific reports. 2017;7(1):13127. Epub 2017/10/17. 10.1038/s41598-017-12419-y . 29030560 PMC5640648

[28] França NAG, Peters BSE, Lima MMS, Santos EA, Santos PC, Martini LA. Muscle mass as the main component of body composition associated with bone mineral density. Osteoporosis International. 2017;28:S210–S1.

[29] Harris R, Chang Y, Beavers K, Laddu-Patel D, Bea J, Johnson K, et al. Risk of Fracture in Women with Sarcopenia, Low Bone Mass, or Both. Journal of the American Geriatrics Society. 2017;65(12):2673–8. Epub 2017/09/30. 10.1111/jgs.15050 . 28960230 PMC5729083

[30] Hwang JA, Kim YS, Leem AY, Park MS, Kim SK, Chang J, et al. Clinical Implications of Sarcopenia on Decreased Bone Density in Men With COPD. Chest. 2017;151(5):1018–27. Epub 2016/12/26. 10.1016/j.chest.2016.12.006 . 28012805

[31] Krajewska-Wlodarczyk M, Owczarczyk-Saczonek A, Placek W. Changes in body composition and bone mineral density in postmenopausal women with psoriatic arthritis. Reumatologia. 2017;55(5):215–21. 10.5114/reum.2017.71627 29332959 PMC5746631

[32] Lee DW, Jin HJ, Shin KC, Chung JH, Lee HW, Lee KH. Presence of sarcopenia in asthma-COPD overlap syndrome may be a risk factor for decreased bone-mineral density, unlike asthma: Korean National Health and Nutrition Examination Survey (KNHANES) IV and V (2008–2011). International journal of chronic obstructive pulmonary disease. 2017;12:2355–62. Epub 2017/08/30. 10.2147/COPD.S138497 . 28848336 PMC5557102

[33] Di Monaco M, Castiglioni C, Bardesono F, Milano E, Massazza G. Sarcopenia, osteoporosis and the burden of prevalent vertebral fractures: a cross-sectional study of 350 women with hip fracture. European journal of physical and rehabilitation medicine [Internet]. 2 12, 2020 Feb 12. https://www.ncbi.nlm.nih.gov/pubmed/32052946. 32052946 10.23736/S1973-9087.20.05991-2

[34] Maurel DB, Jahn K, Lara-Castillo N. Muscle-bone crosstalk: emerging opportunities for novel therapeutic approaches to treat musculoskeletal pathologies. Biomedicines. 2017;5(4):E62. 10.3390/biomedicines5040062 . 29064421 PMC5744086

[35] Lima RM, de Oliveira RJ, Raposo R, Neri SGR, Gadelha AB. Stages of sarcopenia, bone mineral density, and the prevalence of osteoporosis in older women. Archives of osteoporosis. 2019;14(1):38. Epub 2019/03/15. 10.1007/s11657-019-0591-4 . 30868338

